# Phylogeny and evolution of Asparagaceae subfamily Nolinoideae: new insights from plastid phylogenomics

**DOI:** 10.1093/aob/mcac144

**Published:** 2022-11-26

**Authors:** Yunheng Ji, Jacob B Landis, Jin Yang, Shuying Wang, Nian Zhou, Yan Luo, Haiyang Liu

**Affiliations:** CAS Key Laboratory for Plant Diversity and Biogeography of East Asia, Kunming Institute of Botany, Chinese Academy of Sciences, Kunming, Yunnan 650201, China; Yunnan Key Laboratory for Integrative Conservation of Plant Species with Extremely Small Population, Kunming Institute of Botany, Chinese Academy of Sciences, Kunming, Yunnan 650201, China; School of Integrative Plant Science, Section of Plant Biology and the L. H. Bailey Hortorium, Cornell University, Ithaca, NY 14850, USA; BTI Computational Biology Center, Boyce Thompson Institute, Ithaca, NY 14853, USA; CAS Key Laboratory for Plant Diversity and Biogeography of East Asia, Kunming Institute of Botany, Chinese Academy of Sciences, Kunming, Yunnan 650201, China; CAS Key Laboratory for Plant Diversity and Biogeography of East Asia, Kunming Institute of Botany, Chinese Academy of Sciences, Kunming, Yunnan 650201, China; CAS Key Laboratory for Plant Diversity and Biogeography of East Asia, Kunming Institute of Botany, Chinese Academy of Sciences, Kunming, Yunnan 650201, China; University of Chinese Academy of Sciences, Beijing 100049, China; Southeast Asia Biodiversity Research Institute, Chinese Academy of Sciences & Center for Integrative Conservation, Xishuangbanna Tropical Botanical Garden, Chinese Academy of Sciences, Mengla, Yunnan 666303, China; State Key Laboratory of Phytochemistry and Plant Resources in West China, Kunming Institute of Botany, Chinese Academy of Sciences, Kunming, Yunnan 650201, China

**Keywords:** Ancient hybridization, Convallarieae, incomplete lineage sorting, plastome, phylogeny, Polygonateae, *Theropogon*

## Abstract

**Background and aims:**

Asparagaceae subfamily Nolinoideae is an economically important plant group, but the deep relationships and evolutionary history of the lineage remain poorly understood. Based on a large data set including 37 newly sequenced samples and publicly available plastomes, this study aims to better resolve the inter-tribal relationships of Nolinoideae, and to rigorously examine the tribe-level monophyly of Convallarieae, Ophiopogoneae and Polygonateae.

**Methods:**

Maximum likelihood (ML) and Bayesian inference (BI) methods were used to infer phylogenetic relationships of Nolinoideae at the genus level and above. The diversification history of Nolinoideae was explored using molecular dating.

**Key results:**

Both ML and BI analyses identically recovered five clades within Nolinoideae, respectively corresponding to Dracaeneae + Rusceae, Polygonateae + *Theropogon*, Ophiopogoneae, Nolineae, and Convallarieae excluding *Theropogon*, and most deep nodes were well supported. As *Theropogon* was embedded in Polygonateae, the plastome phylogeny failed to resolve Convallarieae and Polygonateae as reciprocally monophyletic. Divergence time estimation showed that the origins of most Nolinoideae genera were dated to the Miocene and Pliocene. The youthfulness of Nolinoideae genera is well represented in the three herbaceous tribes (Convallarieae, Ophiopogoneae and Polygonateae) chiefly distributed in temperate areas of the Northern Hemisphere, as the median stem ages of all 14 genera currently belonging to them were estimated at <12.37 Ma.

**Conclusions:**

This study recovered a robust backbone phylogeny, providing new insights for better understanding the evolution and classification of Nolinoideae. Compared with the deep relationships recovered by a previous study based on transcriptomic data, our data suggest that ancient hybridization or incomplete lineage sorting may have occurred in the early diversification of Nolinoideae. Our findings will provide important reference for further study of the evolutionary complexity of Nolinoideae using nuclear genomic data. The recent origin of these herbaceous genera currently belonging to Convallarieae, Ophiopogoneae and Polygonateae provides new evidence to support the hypothesis that the global expansion of temperate habitats caused by the climate cooling over the past 15 million years may have dramatically driven lineage diversification and speciation in the Northern Hemisphere temperate flora.

## INTRODUCTION

Advancements in molecular phylogenetics over the past few decades have driven extensive taxonomic revision in angiosperms, resulting in great changes in the boundaries of many families (Angiosperm Phylogeny Group, 1998, 2003, 2009, 2016). This is well represented in the monocotyledonous family Asparagaceae (Asparagales): inferences from molecular phylogenies (Chase *et al*., 1995, 2006, 2009; Fay *et al*., 2000; Rudall *et al*., 2000) have dramatically expanded Asparagaceae to accommodate numerous families that were previously circumscribed based on morphological characteristics (Chase *et al*., 2009). As a result, Asparagaceae *sensu*Angiosperm Phylogeny Group (2009, 2016), consisting of approximately 153 genera and 2900 species distributed across both the Old and New Worlds (Stevens, 2001), has become a morphologically diverse and species-rich family of Asparagales.

Asparagaceae is a plant group with significant economic importance. Numerous members of the family have medicinal properties (e.g. *Asparagus*, *Dracaena* and *Polygonatum*), have ornamental value (e.g. *Convallaria*, *Hosta*, *Nolina*, *Ophiopogon* and *Ruscus*), and are used as industrial raw materials due to their high fibre and starch contents (e.g. *Agava* and *Yucca*). The most recent classification of Asparagaceae (Chase *et al*., 2009) divided the family into seven subfamilies (Agavoideae, Aphyllanthoideae, Asparagoideae, Brodiaeoideae, Lomandroideae, Nolinoideae and Scilloideae), and previous phylogenetic studies consistently resolved each as a well-supported monophyletic lineage (Kim *et al*., 2010; Seberg *et al.*, 2012; Steele *et al*., 2012; Chen *et al*., 2013). Among them, the components of Nolinoideae, which combines four families (Convallariaceae, Dracaenaceae, Nolinaceae and Ruscaceae) recognized by Dahlgren *et al*. (1985), exhibit high levels of heterogeneity in composition (Chase *et al*., 2009). As a result, the subfamily possesses considerable floral and vegetative diversity, leading to a lack of morphological synapomorphies that enables distinction from other Asparagaceae subfamilies (Chase *et al*., 2009; Meng *et al*., 2021).

Asparagaceae subfamily Nolinoideae, encompassing about 23 genera, is further divided into seven tribes, namely Convallarieae, Dracaeneae, Eriospermeae, Nolineae, Ophiopogoneae, Polygonateae and Rusceae (Stevens, 2001). Although Eriospermeae has consistently been resolved as sister to the rest of Nolinoideae with strong support, relationships within the remaining Nolinoideae remain poorly resolved (Rudall *et al*., 2000; Jang and Pfosser, 2002; Kim *et al*., 2010; Seberg *et al.*, 2012; Steele *et al*., 2012; Chen *et al*., 2013; Meng *et al*., 2021). Additionally, uncertainty remains regarding whether Convallarieae, Ophiopogoneae and Polygonateae are monophyletic lineages, given that the intergeneric relationships of the three tribes, which are perennially rhizomatous herbs chiefly distributed in temperate areas of Eurasia and North America (Rudall *et al*., 2000), remain contentious (Rudall *et al*., 2000; Yamashita and Tamura, 2000; Kim *et al*., 2010; Seberg *et al.*, 2012; Chen *et al*., 2013; Wang *et al*., 2014; Floden and Schilling, 2018; Meng *et al*., 2021).

Chen *et al*. (2013) proposed that Nolinoideae may represent a recently diversified clade. Due to insufficient phylogenetic information, reconstructing robust phylogenetic trees using single or a few sequence regions is difficult, particularly for plant lineages that have experienced rapid diversification (Rokas and Carroll, 2005; Whitfield and Lockhart, 2007; Philippe *et al*., 2011). Under these circumstances, employing alternative sequence data sets with more informative loci to reconstruct a robust phylogeny of Nolinoideae is necessary. Although Meng *et al*. (2021) used a transcriptomic data set to investigate deep relationships of Nolinoideae and which greatly improved our understanding of this phylogenetically problematic plant group, taxonomic sampling at the genus level within Convallarieae, Ophiopogoneae and Polygonateae was too low to satisfactorily address the issues of tribe-level monophyly or intergeneric relationships of these three herbaceous tribes.

With advancements in high-throughput DNA sequencing technologies, plastid genomes (plastomes), as well as genome-wide nuclear sequence data, have been increasingly used to infer phylogenetic relationships. In contrast to biparentally inherited nuclear genomes, phylogenetic analyses of uniparentally inherited plastomes usually recover only the maternal (or in some cases the paternal) evolutionary history rather than the complete relationships of the lineage. Nevertheless, plastid phylogenomic studies have provided valuable insights into the resolution of historically difficult problems in plant phylogenetics (e.g. Jansen *et al*., 2007; Moore *et al*., 2007, 2010; Parks *et al*., 2009; Huang *et al.*, 2016; Carlsen *et al*., 2018; Ji *et al.*, 2019*a*, 2021; Li *et al*., 2019; Yang *et al.*, 2019). As plastomes have become widely used in phylogenetic studies, previously undetected conflicts between plastid and nuclear phylogenies (cytonuclear discordance) have been found in more plant lineages, providing crucial evidence for inferring complicated evolutionary events, such as incomplete lineage sorting (ILS) and hybridization (e.g. Folk *et al*., 2017; Morales-Briones *et al*., 2018; Ji *et al.*, 2019*a*, 2019*b*; Stull *et al*., 2020; Wen *et al*., 2021; Li *et al*., 2022). Accordingly, plastomes are no less important for phylogenetic reconstruction than nuclear genome data sets and will continue to play an integral role in plant phylogenetics. Based on phylogenomic analyses of a large plastome data set including representatives from 18 out of the 23 genera currently accepted in Nolinoideae, the primary objectives of the present study are: (1) to better resolve the evolutionary relationships among the tribes Convallarieae, Dracaeneae, Nolineae, Ophiopogoneae, Polygonateae and Rusceae; and (2) to rigorously examine the tribe-level monophyly of Convallarieae, Ophiopogoneae and Polygonateae.

## MATERIALS AND METHODS

### Taxon sampling, shotgun sequencing, plastome assembly and annotation

A total of 88 plastomes from 80 species were sampled, including representatives of the six tribes (Convallarieae, Dracaeneae, Nolineae, Ophiopogoneae, Polygonateae and Rusceae) of Asparagaceae subfamily Nolinoideae. Among them, 37 plastomes were newly sequenced in this study (voucher information is presented in Supplementary Data Table S1), and the rest were obtained from the NCBI GenBank database (Table S2, last accessed 22 October 2022). Taxon sampling representing 18 out of the 23 genera of Asparagaceae subfamily Nolinoideae completely covering the currently accepted genera in Convallarieae, Ophiopogoneae and Polygonateae allows for critically exploring intergeneric relationships and testing for monophyly of the three herbaceous tribes.

Genomic DNA of newly collected samples was extracted from silica gel-dried leaf tissue using the CTAB method (Doyle and Doyle, 1987). Shotgun libraries with an average insert size of ~400 bp were constructed using a TruSeq DNA PCR-free prep kit (Illumina Inc., San Diego, CA, USA) following the manufacturer’s instruction. Prepared libraries were sequenced on an Illumina Novaseq 6000. For each sample, paired-end sequencing (2 × 150 bp) generated ~4 Gb of raw reads, and Trimmomatic v0.40 (Bolger *et al.*, 2014) was used to remove adaptors and to filter low-quality reads with preset parameters. The GetOrganelle v1.7.5.0 pipeline (Jin *et al*., 2020) was used to recover plastomes from filtered Illumina sequencing reads with default parameters, using the complete plastome of *Dracaena hokouensis* (GenBank accession number: MN200197) as the reference. The assembled plastomes were annotated using the Plastid Genome Annotator (Qu *et al*., 2019) and further validated by performing a BLAST search against the NCBI protein data set with Geneious v10.2.3 (Kearse *et al*., 2012). The junctions of the large-single copy (LSC), small-single copy (SSC) and inverted-repeat (IR) regions for each plastome were visually examined and manually adjusted by comparison with the reference plastome using Geneious v10.2.3 (Kearse *et al*., 2012).

### Phylogenomic analyses

In addition to representatives of Asparagaceae subfamily Nolinoideae, 35 publicly available plastomes (Supplementary Data Table S2) representing five Asparagales families (Amaryllidaceae, Asphodelaceae, Hypoxidaceae, Iridaceae and Orchidaceae) and the remaining Asparagaceae subfamilies (Agavoideae, Aphyllanthoideae, Asparagoideae, Brodiaeoideae, Lomandroideae and Scilloideae) were incorporated into the data set. Given the close relationship between Asparagales and Liliales (Givnish *et al*., 2018), six taxa from three Liliales families (Colchicaceae, Liliaceae and Melanthiaceae) were selected as the outgroup. Among the sampled plastomes, 68 commonly shared plastid protein-coding genes (PCGs) were extracted from the complete plastome data set using the software PhyloSuite v1.1.15 (Zhang *et al*., 2020). The PCGs were aligned and concatenated with MAFFT v7.402 (Katoh and Standley, 2013) using default parameters. The best partitioning schemes for the concatenated data set were determined using PartitionFinder v2.1.1 (Lanfear *et al*., 2017), using the ModelFinder (Kalyaanamoorthy *et al*., 2017) option to identify the optimal partitioning scheme and substitution models for among-site rate heterogeneity.

Based on the recommended partitioning schemes and substitution models, phylogenetic analyses were performed using both maximum likelihood (ML) and Bayesian inference (BI) methods. The ML phylogeny was reconstructed with RAxML-HPC BlackBox v8.1.24 (Stamatakis, 2006), estimating the support value for each node with 1000 bootstrap (BS) replicates. The BI phylogeny was inferred using MrBayes v3.2 (Ronquist *et al*., 2012). BI analysis comprised two simultaneous and independent Markov chain Monte Carlo (MCMC) runs of 10 million generations, sampling one tree every 1000 generations with the first 25 % of trees abandoned as burn-in. After reaching the stationary state when the average standard deviation of the split frequencies was <0.01, the two independent runs were combined to obtain the majority rule consensus trees and to calculate posterior probabilities (PP).

### Estimation of divergence times

Based on the concatenated data set of 68 plastid PCGs, divergence times were estimated with BEAST v2.4.7 (Bouckaert *et al*., 2014). The molecular clock was calibrated with the incorporation of six secondary calibration priors provided by a previous study (Givnish *et al*., 2018): (1) 116.32 million years ago (Ma) for the crown age of Asparagales; (2) 68.72 Ma for the stem age of Iridaceae; (3) 59.38 Ma for the stem age of Asphodelaceae; (4) 52.09 Ma for the divergence between Amaryllidaceae and Asparagaceae; (5) 49.53 Ma for the crown age of Asparagaceae; and (6) 43.12 Ma for the divergence between Asparagaceae subfamilies Asparagoideae and Nolinoideae. We used the ML tree as a topological constraint in the BEAST analysis, with the uncorrelated log-normal relaxed clock approach with a Yule tree prior, and under the sequence substitution models recommended by PartitionFinder. The MCMC simulations were run for 500 million generations, sampling a tree every 5000 generations with the first 10 % of trees being discarded as burn-in. The convergence of the MCMC stimulations was inspected in TRACER v1.7.1 (Rambaut *et al*., 2018), and the maximum clade credibility tree with median ages and 95 % highest posterior density (HPD) intervals for all nodes was visualized in FIGTREE v1.3.1 (http://tree.bio.ed.ac.uk/software/figtree/).

## RESULTS

### Illumina sequencing, plastome assembly and characteristics

A summary of Illumina sequencing and plastome assembly is presented in Supplementary Data Table S3: the reference-guided plastome assembly recovered the complete plastomes of all samples with sequence coverage ranging from 113.727× to 1256.114× (Table S3). These newly sequenced plastomes were deposited in NCBI GenBank with the accession numbers shown in Table S1. The newly sequenced plastomes varied from 153 883 to 162 227 bp in size, with the GC content ranging from 37.3 to 38.0 % (Table S4). Except for loss of the *rps16* gene in the plastome of *Ruscus aculeatus*, each plastome identically possessed 114 unique genes, including 80 PCGs, 30 tRNA genes and four plastid rRNA genes (Table S5). Additionally, an insertion of ~3.3 kb in the IR regions was found in the plastomes of *Convallaria majalis*. This mutation was also observed in its congeneric species, *C. keiskei*, and was proposed to be caused by horizontal gene transfer between mitochondrial and plastid genomes (Raman *et al*., 2019).

### Phylogenetic relationships

**Fig. 1.**
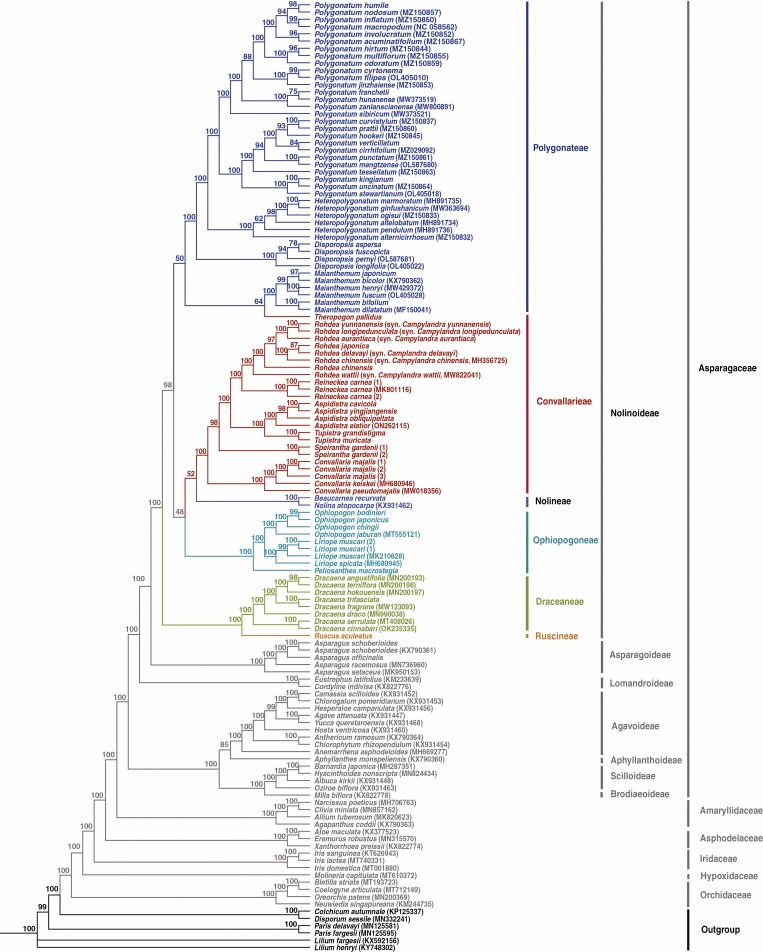
Phylogeny of Asparagaceae reconstructed by analyses of 68 plastid protein-coding genes (PCGs) using the maximum likelihood (ML) method. Numbers above branches indicate ML bootstrap (BS) percentages.

**Fig. 2.**
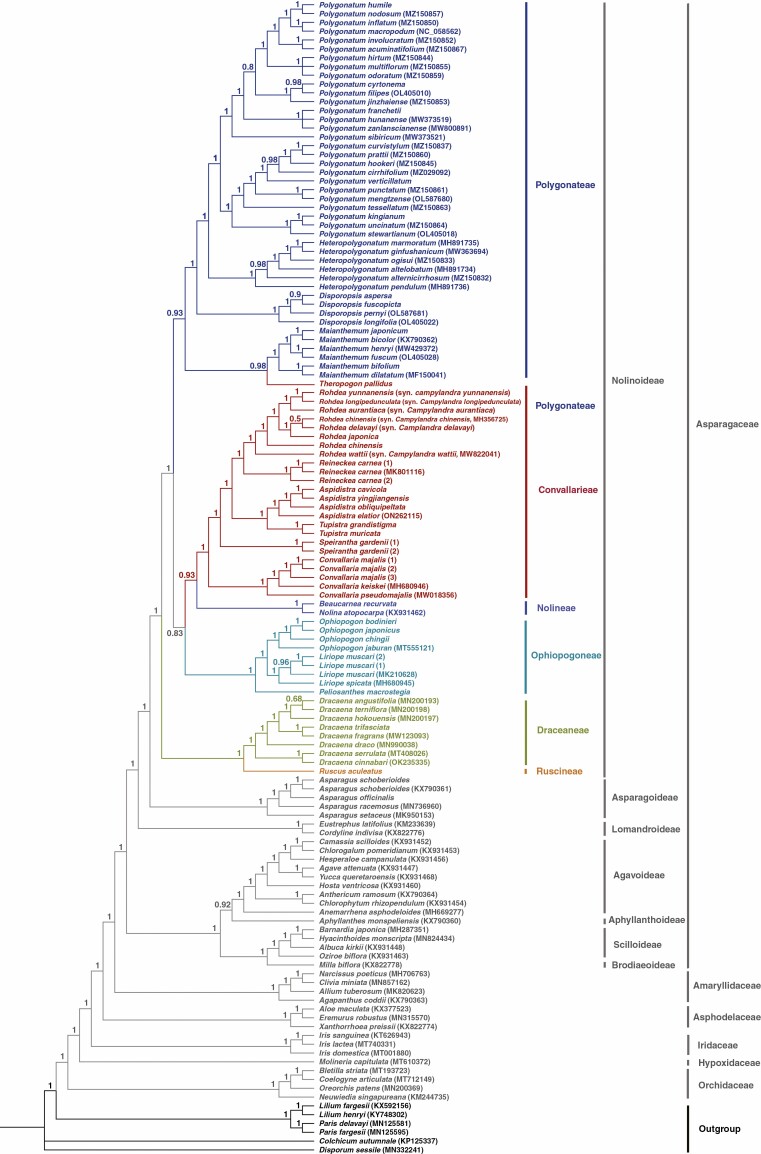
Phylogeny of Asparagaceae reconstructed by analyses of 68 plastid protein-coding genes (PCGs) using the Bayesian inference (BI) method. Numbers above branches indicate BI posterior probability (PP).

The concatenated matrix of 68 plastid PCGs was 65 859 bp in length, including 21 994 variable sites, of which 16 068 were parsimony-informative (Supplementary Data Table S6). Based on the concatenated matrix, the ML and BI phylogenies were almost identical in tree topologies, despite several nodes recovered with low support values in the ML phylogeny (Fig. 1) were well supported in the BI phylogeny (Fig. 2). All seven subfamilies of Asparagaceae outlined by Chase *et al*. (2009) were recovered as monophyletic and grouped in two well-supported major clades (BS = 100 %, PP = 1.00), within which the successive divergence of Brodiaeoideae + Scilloideae, Aphyllanthoideae and Agavoideae, as well as of Lomandroideae, Asparagoideae and Nolinoideae, were recovered. Except for the sister relationship between Aphyllanthoideae and Agavoideae (BS = 85 %, PP = 0.92), all nodes at the subfamily level were fully supported (BS = 100 %, PP = 1.00).

Within Asparagaceae subfamily Nolinoideae, our plastid phylogenomic analyses identically recovered five clades corresponding to (1) Dracaeneae + Rusceae (BS = 100 %, PP = 1.00), (2) Polygonateae + *Theropogon* (BS = 50 %, PP = 0.93), (3) Ophiopogoneae (BS = 100 %, PP = 1.00), (4) Nolineae (BS = 100 %, PP = 1.00) and (5) Convallarieae excluding *Theropogon* (BS = 100 %, PP = 1.00). Due to *Theropogon* being embedded in Polygonateae, both Convallarieae and Polygonateae were not resolved as reciprocally monophyletic by either the ML or BI phylogenies. In Convallarieae (excluding *Theropogon*), a highly resolved and well-supported intergeneric phylogeny was recovered: *Convallaria* was resolved as the earliest diverging clade, which was successively sister to *Speirantha* (BS = 98 %, PP = 1.00), *Tupistra* + *Aspidistra* (BS = 100 %, PP = 1.00), *Reineckea* (BS = 100 %, PP = 1.00) and *Rohdea* (BS = 100 %, PP = 1.00). In Ophiopogoneae, successive divergences of *Peliosanthes*, *Liriope* and *Ophiopogon* were recovered with robust branch support (BS = 100 %, PP = 1.00). The Polygonateae + *Theropogon* clade was resolved as two subclades. The first comprised *Theropogon* and *Maianthemum* (BS = 64 %, PP = 0.98); within the second subclade, *Polygonatum* was sister to *Heteropolygonatum* (BS = 100 %, PP = 1.00), and the two genera, in turn, were sister to *Disporopsis* (BS = 100 %, PP = 1.00).

### Molecular dating

**Fig. 3.**
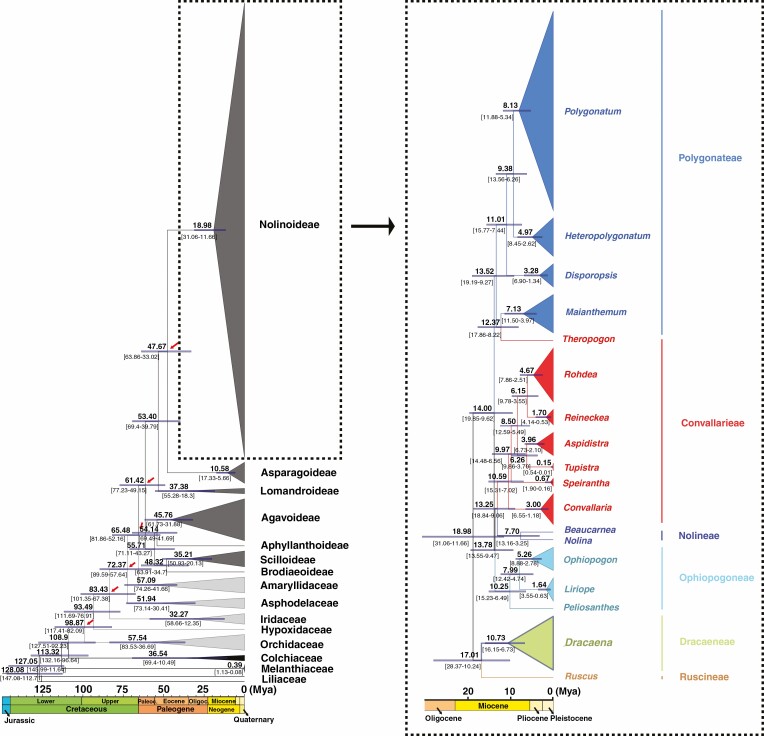
Divergence time estimation based on 68 plastid protein-coding genes. Numbers above and below the branches represent mean divergence ages and 95 % confidence interval of each node. Red arrows show the calibration points for molecular dating. Divergence time and the timeline are indicated in million years ago.

Among the five successively divergent clades recovered in Asparagaceae subfamily Nolinoideae, the early divergence of the Dracaeneae + Rusceae clade occurred ~18.89 Ma, and Polygonateae + *Theropogon* clade emerged at ~14.00 Ma followed by the divergence of Ophiopogoneae at ~13.78 Ma (Fig. 3). Subsequently, Nolineae diverged from Convallarieae (excluding *Theropogon*) at ~13.25 Ma (Fig. 3). The crown ages of the five successive diverging clades were dated to ~17.01 Ma (Cracaeneae + Rusceae), ~13.52 Ma (Polygonateae + *Theropogon*), ~10.25 Ma (Ophiopogoneae), ~10.59 Ma (Convallarieae excluding *Theropogon*) and ~7.70 Ma (Nolineae), respectively (Fig. 3).

## DISCUSSION

### Relationships among Asparagaceae subfamilies

Due to its great economic importance, the phylogeny of Asparagaceae has been extensively investigated (e.g. Chase *et al*., 1995, 2006; Fay *et al*., 2000; Rudall *et al*., 2000; Kim *et al*., 2010; Seberg *et al.*, 2012; Steele *et al*., 2012; Chen *et al*., 2013), with previous studies identically resolving the seven subfamilies circumscribed by Chase *et al*. (2009) as well-supported monophyletic lineages (Pires *et al*., 2006; Seberg *et al.*, 2012; Steele *et al*., 2012; Chen *et al*., 2013). The relationships among them, however, remain poorly resolved. This is mainly due to the ambiguous position of Aphyllanthoideae, which has been proposed to be sister to Agavoideae (Seberg *et al.*, 2012; Steele *et al*., 2012), Brodiaeoideae (Chen *et al*., 2013) and Lomandroideae (Pires *et al*., 2006).

Our phylogenomic analyses not only recovered the seven subfamilies as monophyletic but also provided robust support for their relationships. At the subfamily level, the relationships recovered in this study are identical to that inferred from the combination of plastid, mitochondrial and nuclear ribosomal gene data sets (Steele *et al.* 2012), but with quite strong support for each node. Our results further confirm the sister relationship of Aphyllanthoideae and Agavoideae, and suggest that Agavoideae, Aphyllanthoideae, Brodiaeoideae and Scilloideae may have originated from a common maternal ancestor. This study recovered a robust backbone phylogeny of Asparagaceae at the subfamily level, providing new insights for elucidating the long-standing controversies over the deep phylogenetic relationships of this economically important plant group.

### Phylogeny and evolution of Nolinoideae

Nolinoideae is a phylogenetically problematic subfamily within Asparagaceae, given that the tribe-level relationships (except for the early divergence of Eriospermeae) and the monophyly of Convallarieae, Ophiopogoneae and Polygonateae remain unresolved. In this study, except for *Comospermum*, *Danae*, *Dasylirion*, *Eriospermum* and *Semele*, representatives of 18 out of the 23 genera currently accepted in Nolinoideae were included in the phylogenetic analyses. Based on the comprehensive taxonomic sampling and the concatenated 68 plastid PCG data set that contains more variable sites and parsimony-informative variations than was available in previous studies, this study provides new insights for better understanding the relationships of phylogenetically problematic lineages at the genus level and above.

**Fig. 4.**
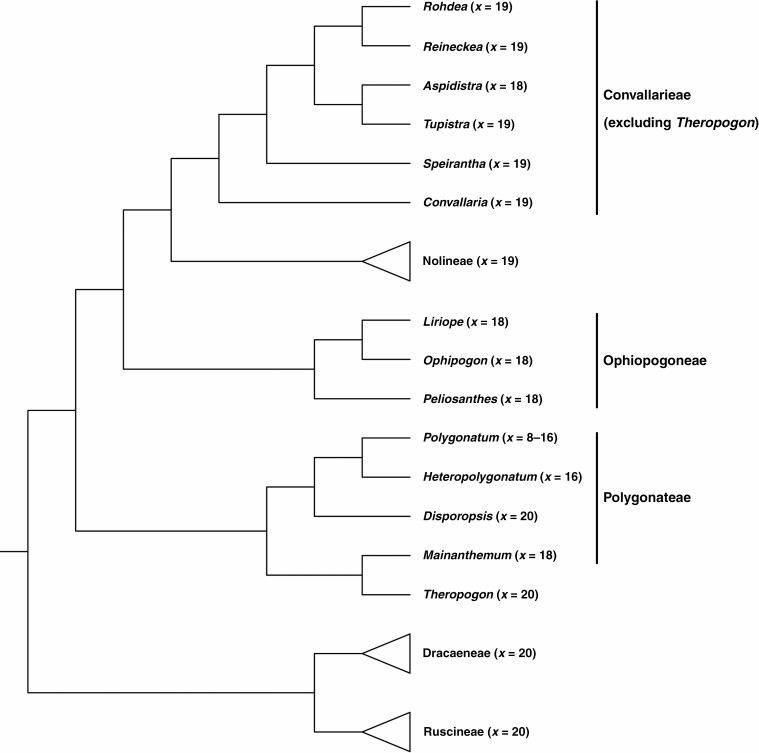
Basic chromosome number at the genus level mapped along the plastome phylogeny of Nolinoideae.

**Fig. 5.**
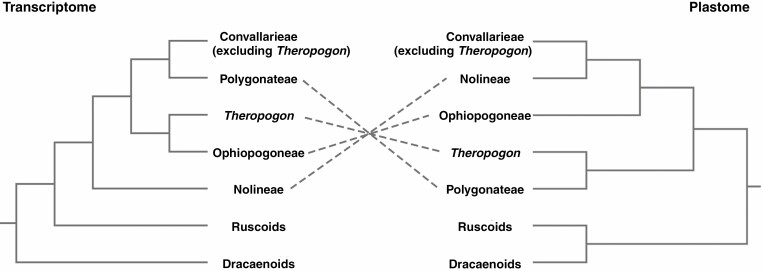
Comparison of deep relationships of Nolinoideae recovered from analyses of transcriptomic data (Meng *et al*., 2021) and plastomes (this study).

The close relationships between Ruscineae and Convallarieae (Rudall *et al*., 2000), as well as between Dracaeneae and Nolineae (Kim *et al*., 2010), were proposed by previous studies. However, our plastid phylogenomic analyses showed Ruscineae is more closely related to Dracaeneae than to Convallarieae (excluding *Theropogon*), and Nolineae is closely allied to Convallarieae (excluding *Theropogon*) rather than to Dracaeneae. Notably, the close relationships between Dracaeneae and Ruscineae (Chen *et al*., 2013; Meng *et al*., 2021), as well as between Convallarieae (excluding *Theropogon*) and Nolineae (Seberg *et al.*, 2012), were also proposed by previous studies based on different sequence data sets. These affinities can be justified by cytological evidence (Fig. 4), given that Dracaeneae and Ruscineae have a basic chromosome number *x* = 20, in contrast to the basic chromosome number *x* = 19 of Convallarieae (excluding *Theropogon*) and Nolineae. Nevertheless, based on transcriptome data, high levels of gene tree conflict regarding the relationships of Nolineae, Ophiopogoneae, Polygonateae and *Theropogon* with other Nolinoideae were detected, and such discordance was hypothesized to have been caused by ancient hybridization or ILS (Meng *et al*., 2021). Since the close relationships between Convallarieae (excluding *Theropogon*) and Polygonateae, Ophiopogoneae and *Theropogon*, as well as Nolineae and the clade consisting of the three herbaceous tribes were strongly supported by phylogenomic analyses of transcriptome data (Meng *et al*., 2021), the relationships are quite different from those recovered by our data (Fig. 5). In addition to the nuclear gene tree conflict identified by Meng *et al*. (2021), there is also large discordance regarding the phylogenetic positions of Nolineae, Ophiopogoneae, Polygonateae and *Theropogon* between the transcriptome (Meng *et al*., 2021) and plastome (this study) tree topologies, providing good support to the hypothesis that the early evolution of these taxa may have undergone hybridization or ILS (Meng *et al*., 2021).

Regarding the three herbaceous tribes (Convallarieae, Ophiopogoneae and Polygonateae) within Nolinoideae, the non-monophyly of Convallarieae has been generally revealed in previous studies (Kim *et al*., 2010; Seberg *et al*., 2012; Chen *et al*., 2013; Meng *et al*., 2021). By contrast, only a few studies resolved Ophiopogoneae (Jang and Pfosser, 2002; Seberg *et al.*, 2012) and Polygonateae (Seberg *et al.*, 2012; Floden and Schilling, 2018; Wang *et al.*, 2022) as non-monophyletic, and most studies support the monophyly of Ophiopogoneae (Rudall *et al*., 2000; Yamashita and Tamura, 2000; Kim *et al*., 2010; Wang *et al.*, 2014; Floden and Schilling, 2018) and Polygonateae (Rudall *et al*., 2000; Yamashita and Tamura, 2000; Meng *et al*., 2008, 2014; Wang *et al.*, 2022). Notably, previous studies based on genome-scale sequence data (e.g. Floden and Schilling, 2018; Meng *et al*., 2021; Wang *et al.*, 2022) had limited generic sampling from the three herbaceous tribes, which may have resulted in phylogenetic errors or uncertainty in the tree topology (Rokas and Carroll, 2005; Philippe *et al*., 2011), and consequently led to ambiguity on the monophyletic nature of the three herbaceous tribes.

With a complete generic sampling of the three herbaceous tribes, our results showed that Ophiopogoneae is a well-supported monophyletic lineage. Consistent with morphological characteristics, the three genera (*Liriope*, *Ophiopogon* and *Peliosanthes*) traditionally assigned to Ophiopogoneae share the unusual morphologies that their capsules dehisce early to expose the immature seeds during development (Jessop, 1976) and their basic chromosome number is *x* = 19 (Rudall *et al*., 2000). Accordingly, recognizing Ophiopogoneae as a distinctive tribe is reasonable (Conran, 1989; Wang *et al*., 2014) rather than to place *Peliosanthes* in a separate tribe, Peliosantheae (Nakai, 1936; Dai and Liang, 1991; Liang and Dai, 1992). Although Mcharo *et al*. (2003) and Yamashita and Tamura (2004) proposed that *Liriope* is closely related to *Peliosanthes* but disparate from *Ophiopogon*, the present study proposes that *Liriope* has a sister relationship with *Ophiopogon*, and these two genera, in turn, are sister to *Peliosanthes*. The well-supported intergeneric relationships of Ophiopogoneae revealed by our data can be further restrengthened by previous studies (Rudall *et al*., 2000; Floden and Schilling, 2018), which generated identical results, as well as by morphological and palynological evidence (Chang and Hsu, 1974; Dai and Liang, 1991; Cutler, 1992; Liang and Dai, 1992; Rudall *et al*., 2000).

On the other hand, our plastid phylogenomic analyses recovered neither Convallarieae nor Polygonateae as monophyletic. As the tree topology indicated, *Theropogon* is phylogenetically disparate from the rest of Convallarieae but closely related to *Maianthemum*, and the two genera are sister to the clade including the remaining genera (*Disporopsis*, *Heteropolygonatum* and *Polygonatum*) of Polygonateae. Additionally, without the inclusion of *Theropogon*, the rest of Convallarieae formed a well-supported clade. The relationships are consistent with some morphological features. Specifically, *Theropogon* possesses ovarian nectaries, which resembles Polygonateae but differs from the absence of a flora nectary in the rest of Convallarieae (Vaikos *et al*., 1989). This supports the exclusion of *Theropogon* from Convallarieae and as a lineage closer to Polygonateae. Additionally, the stamens are free in *Maianthemum* and *Theropogon* but are adnate to tepals in the remaining genera of Convallarieae and Polygonateae (Rudall *et al*., 2000), which supports the close relationship between *Maianthemum* and *Theropogon*. Taken together, the reciprocally reinforcing evidence suggests that taxonomic work based on multidisciplinary data is needed to establish the monophyly of Convallarieae and Polygonateae.

With the exclusion of *Theropogon*, this study recovered a well-supported intergeneric phylogeny for the rest of Convallarieae, and resolved *Convallaria* and *Speirantha* as two early diverging lineages of the clade. The placement of these two genera can be justified based on some morphological features: they both possess underground rhizomes and long and slender creeping stems, unlike *Aspidistra*, *Rohdea, Tupistra*, and *Reineckia*, which have prostrate or ascending rhizomes above the ground and extremely shortened (or nearly absent) stems, respectively, providing support for the close relationship between *Convallaria* and *Speirantha*; as *Convallaria* is distinctive in having nodding flowers in contrast to the erect flowers of *Aspidistra*, *Rohdea*, *Reineckia*, *Speirantha* and *Tupistra*, this supports the transitional position of *Speirantha* between *Convallaria* and the subclade consisting of *Rohdea*, *Reineckia*, *Speirantha* and *Tupistra*. Additionally, this study also provides insightful evidence to resolve the disagreements over the generic circumscription of *Campylandra* (currently synonymized to *Rohdea*), *Rohdea* and *Tupistra*. Briefly, *Campylandra* and *Tupistra* were recognized as two distinct genera (Baker, 1875; Engler, 1888; Hutchinson, 1934; Liang and Tamura, 2000; Tamura *et al*., 2000), although some authors proposed that they are congeneric (e.g. Bentham, 1883; Hooker, 1892; Liang, 1978). Based on comprehensive morphological analyses, the morphological differences between *Campylandra* and *Rohdea* are unlikely to be robust enough for the recognition of the two as separate genera (Tanaka, 2003); accordingly, Yamashita and Tamura (2004) merged *Campylandra* with *Rohdea*. Our results show that *Rohdea* and *Campylandra* are not reciprocally monophyletic, while *Tupistra* is more closely related to *Aspidistra* than to *Campylandra*. This implies that *Campylandra* is not congeneric with *Tupistra*, and validates the taxonomic proposal that reduced *Campylandra* to a synonym of *Rohdea* (Yamashita and Tamura, 2004).

Previous studies have shown that the origins of some Asparagaceae genera, such as *Agave sensu lato* and allied genera (Good-Avila *et al*., 2006; Flores-Abreu *et al*., 2019; Jiménez-Barron *et al*., 2020), the *Milla* complex (Gándara *et al*., 2014), and *Yucca* (Smith *et al*., 2008), can be traced back to the Miocene or Pliocene. Similarly, our results suggest that the most extensive lineage divergence at the genus level, which resulted in the formation of genera in Asparagaceae subfamily Nolinoideae, took place in the Miocene. The youthfulness of genera is more evident in the three herbaceous tribes (Convallarieae, Ophiopogoneae and Polygonateae) chiefly distributed in the temperate areas of the Northern Hemisphere, as the median stem ages of all the 14 genera currently belonging to them were estimated to <12.37 Ma. The recent radiative divergence of these herbaceous genera is congruent with the speculation that the global expansion of temperate habitats caused by climate cooling over the past 15 million years has contributed greatly to lineage diversification and speciation in the Northern Hemisphere temperate flora (Folk *et al*., 2019; Sun *et al*., 2020).

## CONCLUSIONS

The robust plastome phylogeny reconstructed in this study provides insightful perspectives for better understanding the deep relationships and classification of Nolinoideae, a phylogenetically problematic lineage. The significant incongruences between our plastome phylogeny and previous results from phylogenetic analyses of transcriptomic data (Meng *et al*., 2021) suggests that hybridization or ILS may have occurred in the early diversification of Nolinoideae. The findings provide new insights into the phylogeny and evolution of Nolinoideae. Nevertheless, the taxonomic sampling of Nolinoideae at the genus level in this study is incomplete, due to the absence of five genera, particular the enigmatic *Comospermum*. Additionally, both misspecification of the substitution model for plastid and nuclear data sets and erroneous assembly of polyploid transcriptome data probably result in phylogenetic errors, which may in turn lead to the incongruence between nuclear and plastid phylogenies of Nolinoideae. To critically explore the evolutionary complexity of Nolinoideae, a sampling strategy covering all genera currently recognized in Nolinoideae and the application of nuclear genomic data are needed.

## SUPPLEMENTARY DATA

Supplementary data are available online at https://academic.oup.com/aob and consist of the following. Table S1. Collection information of samples and GenBank accession numbers of newly sequenced plastomes in this study. Table S2: Publicly available plastomes obtained from GenBank. Table S3: Summary of Illumina sequence and plastome assembly. Table S4: Features of Nolinoideae plastomes. Table S5: List of genes identified in Nolinoideae plastomes. Table S6: Sequence characteristics of 68 protein-coding genes involved in the phylogenetic analyses.

mcac144_suppl_Supplementary_Table_S1

mcac144_suppl_Supplementary_Table_S2

mcac144_suppl_Supplementary_Table_S3

mcac144_suppl_Supplementary_Table_S4

mcac144_suppl_Supplementary_Table_S5

mcac144_suppl_Supplementary_Table_S6
