# Supplementary material for: Phylogeny and evolution of Asparagaceae subfamily Nolinoideae: new insights from plastid phylogenomics
Source: Ann Bot. 2022 Nov 26;131(2):301–12. doi: 10.1093/aob/mcac144 (PMC9992941; doi:10.1093/aob/mcac144)
Supplement: mcac144_suppl_Supplementary_Table_S1 [file mcac144_suppl_supplementary_table_s1.docx]

**Table S1. Collection information of samples and GenBank accession of plastomes newly sequenced in this study.**

| **No.** | **Tribe** | **Taxa** | **Voucher** | **Locality** | **GenBank accession** |
| --- | --- | --- | --- | --- | --- |
| 1 | Convallarieae | *Aspidistra cavicola* | Ji Y 2019092 | Cultivated in Kunming Botanical Garden, Yunnan, China | ON872717 |
| 2 | Convallarieae | *Aspidistra obliquipeltata* | B2013-477 | Chongzuo, Guangxi, China | ON872711 |
| 3 | Convallarieae | *Aspidistra yingjiangensis* | Ji Y 2019093 | Cultivated in Kunming Botanical Garden, Yunnan, China | ON872706 |
| 4 | Convallarieae | *Convallaria majalis* (1) | Liu M et al 598 | Shangzhi, Heilongjiang, China | ON872704 |
| 5 | Convallarieae | *Convallaria majalis* (2) | Zhou H 1506 | Antu, Jilin, China | ON872729 |
| 6 | Convallarieae | *Convallaria majalis* (3) | Ji Y 2018168 | Fuyuan, Heilongjiang, China | ON872708 |
| 7 | Convallarieae | *Reineckea carnea* (1) | Ji Y 2019101 | Cultivated in Kunming Botanical Garden, Yunnan, China | ON872715 |
| 8 | Convallarieae | *Reineckea carnea* (2) | An H 2019112 | Wuding, Yunnan, China | ON872727 |
| 9 | Convallarieae | *Rohdea aurantiaca* | Zhou Y 141 | Tengchong, Yunnan, China | ON872705 |
| 10 | Convallarieae | *Rohdea chinensis* | Ji Y 2019087 | Cultivated in Kunming Botanical Garden, Yunnan, China | ON872725 |
| 11 | Convallarieae | *Rohdea delavayi* | 15CS10509 | Qiaojia, Yunnan, China | ON872710 |
| 12 | Convallarieae | *Rohdea japonica* | Dong A et al Tancm966 | Lushan District, Jiangxi, China | ON872703 |
| 13 | Convallarieae | *Rohdea longipedunculata* | Ji Y 2019109 | Cultivated in Kunming Botanical Garden, Yunnan, China | ON872726 |
| 14 | Convallarieae | *Rohdea yunnanensis* | Ji Y 2019086 | Cultivated in Kunming Botanical Garden, Yunnan, China | ON872709 |
| 15 | Convallarieae | *Speirantha gardenii* (1) | Ji Y 2019094 | Cultivated in Kunming Botanical Garden, Yunnan, China | ON872718 |
| 16 | Convallarieae | *Speirantha gardenii* (2) | Ya J 15CS11175 | Xiuning, Anhui, China | ON872696 |
| 17 | Convallarieae | *Theropogon pallidus* | Exp. 4213 | Dinggye, Tibet, China | ON872724 |
| 18 | Convallarieae | *Tupistra grandistigma* | Ji Y 2020111 | Cultivated in Kunming Botanical Garden, Yunnan, China | ON872713 |
| 19 | Convallarieae | *Tupistra muricata* | 13CS6063 | Laos | ON872699 |
| 20 | Dracaeneae | *Dracaena trifasciata* | Jin L 2020051 | Kunming, Yunnan, China | ON872733 |
| 21 | Nolineae | *Beaucarnea recurvata* | Luo Y *s. n.* | Cultivated in Xishuangbanna Tropical Botanical Garden, Yunnan, China | ON872730 |
| 22 | Ophiopogoneae | *Liriope muscari* (1) | Ji Y 2019107 | Cultivated in Kunming Botanical Garden, Yunnan, China | ON872721 |
| 23 | Ophiopogoneae | *Liriope muscari* (2) | Ji Y 2019091 | Cultivated in Kunming Botanical Garden, Yunnan, China | ON872714 |
| 24 | Ophiopogoneae | *Ophiopogon bodinieri* | An H 2019110 | Wuding, Yunnan, China | ON872697 |
| 25 | Ophiopogoneae | *Ophiopogon chingii* | Ji Y 2019088 | Cultivated in Kunming Botanical Garden, Yunnan, China | ON872722 |
| 26 | Ophiopogoneae | *Ophiopogon japonicus* | An H 2019111 | Wuding, Yunnan, China | ON872707 |
| 27 | Ophiopogoneae | *Peliosanthes macrostegia* | LED9297 | Chongzuo, Guangxi, China | ON872701 |
| 28 | Polygonateae | *Disporopsis aspersa* | Ji Y 2019108 | Cultivated in Kunming Botanical Garden, Yunnan, China | ON872728 |
| 29 | Polygonateae | *Disporopsis fuscopicta* | Liu C et al. 12CS4462 | Zhenyuan, Yunnan, China | ON872731 |
| 30 | Polygonateae | *Maianthemum bifolium* | Ji Y 2018163 | Fuyuan, Heilongjiang, China | ON872719 |
| 31 | Polygonateae | *Maianthemum japonicum* | An H 2019002 | Panshi, Heilongjiang, China | ON872716 |
| 32 | Polygonateae | *Polygonatum cyrtonema* | Ji Y 2019077 | Cultivated in Kunming Botanical Garden, Yunnan, China | ON872700 |
| 33 | Polygonateae | *Polygonatum franchetii* | Ji Y 2018222 | Qu County, Sichuan, China | ON872720 |
| 34 | Polygonateae | *Polygonatum humile* | Ji Y 2018115 | Siziwang Banner, Inner Mongolia | ON872732 |
| 35 | Polygonateae | *Polygonatum kingianum* | Ji Y 2019003 | Gengma, Yunnan, China | ON872712 |
| 36 | Polygonateae | *Polygonatum verticillatum* | Yi S 2019128 | Cultivated in Kunming Botanical Garden, Yunnan, China | ON872698 |
| 37 | Ruscineae | *Ruscus aculeatus* | Liu C 2020049 | Cultivated in Kunming Botanical Garden, Yunnan, China | ON872723 |
| 38 | - | *Asparagus officinalis* | Ji Y 2019084 | Cultivated in Kunming Botanical Garden, Yunnan, China | ON872702 |
| 39 | - | *Asparagus schoberioides* | Ji Y 2018152 | Panshi, Jilin, China | ON872695 |
