# Supplementary material for: Phylogeny and evolution of Asparagaceae subfamily Nolinoideae: new insights from plastid phylogenomics
Source: Ann Bot. 2022 Nov 26;131(2):301–12. doi: 10.1093/aob/mcac144 (PMC9992941; doi:10.1093/aob/mcac144)
Supplement: mcac144_suppl_Supplementary_Table_S2 [file mcac144_suppl_supplementary_table_s2.docx]

**Table S2. Publicly available plastomes obtained from GenBank**

| **No.** | **Family** | **Species** | **GenBank accession** |
| --- | --- | --- | --- |
| 1 | Amaryllidaceae | *Agapanthus coddii* | KX790363 |
| 2 | Amaryllidaceae | *Narcissus poeticus* | MH706763 |
| 3 | Amaryllidaceae | *Allium tuberosum* | MK820623 |
| 4 | Amaryllidaceae | *Clivia miniata* | MN857162 |
| 5 | Asparagaceae | *Agave attenuata* | KX931447 |
| 6 | Asparagaceae | *Albuca kirkii* | KX931448 |
| 7 | Asparagaceae | *Anemarrhena asphodeloides* | MH669277 |
| 8 | Asparagaceae | *Anthericum ramosum* | KX790364 |
| 9 | Asparagaceae | *Aphyllanthes monspeliensis* | KX790360 |
| 10 | Asparagaceae | *Asparagus racemosus* | KX790360 |
| 11 | Asparagaceae | *Asparagus schoberioides* | KX790361 |
| 12 | Asparagaceae | *Asparagus setaceus* | MK950153 |
| 13 | Asparagaceae | *Aspidistra elatior* | ON262115 |
| 14 | Asparagaceae | *Barnardia japonica* | MH287351 |
| 15 | Asparagaceae | *Camassia scilloides* | KX931452 |
| 16 | Asparagaceae | *Chlorogalum pomeridianum* | KX931453 |
| 17 | Asparagaceae | *Chlorophytum rhizopendulum* | KX931454 |
| 18 | Asparagaceae | *Convallaria keiskei* | MH680946 |
| 19 | Asparagaceae | *Convallaria pseudomajalis* | MW018356 |
| 20 | Asparagaceae | *Cordyline indivisa* | KX822776 |
| 21 | Asparagaceae | *Disporopsis longifolia* | OL405022 |
| 22 | Asparagaceae | *Disporopsis pernyi* | OL587681 |
| 23 | Asparagaceae | *Dracaena angustifolia* | MN200193 |
| 24 | Asparagaceae | *Dracaena cinnabari* | OK235335 |
| 25 | Asparagaceae | *Dracaena draco* | MN990038 |
| 26 | Asparagaceae | *Dracaena fragrans* | MW123093 |
| 27 | Asparagaceae | *Dracaena hokouensis* | MN200197 |
| 28 | Asparagaceae | *Dracaena serrulata* | MT408026 |
| 29 | Asparagaceae | *Dracaena terniflora* | MN200198 |
| 30 | Asparagaceae | *Eustrephus latifolius* | KM233639 |
| 31 | Asparagaceae | *Hesperaloe campanulata* | KX931456 |
| 32 | Asparagaceae | *Heteropolygonatum altelobatum* | MH891734 |
| 33 | Asparagaceae | *Heteropolygonatum alternicirrhosum* | MZ150832 |
| 34 | Asparagaceae | *Heteropolygonatum ginfushanicum* | MW363694 |
| 35 | Asparagaceae | *Heteropolygonatum marmoratum* | MH891735 |
| 36 | Asparagaceae | *Heteropolygonatum ogisui* | MZ150833 |
| 37 | Asparagaceae | *Heteropolygonatum pendulum* | MH891736 |
| 38 | Asparagaceae | *Hosta ventricosa* | KX931460 |
| 39 | Asparagaceae | *Hyacinthoides non-scripta* | MN824434 |
| 40 | Asparagaceae | *Liriope muscari* | MK210628 |
| 41 | Asparagaceae | *Liriope spicata* | MH680945 |
| 42 | Asparagaceae | *Maianthemum bicolor* | KX790362 |
| 43 | Asparagaceae | *Maianthemum dilatatum* | MF150041 |
| 44 | Asparagaceae | *Maianthemum fuscum* | OL405028 |
| 45 | Asparagaceae | *Maianthemum henryi* | MW429372 |
| 46 | Asparagaceae | *Milla biflora* | KX822778 |
| 47 | Asparagaceae | *Nolina atopocarpa* | KX931462 |
| 48 | Asparagaceae | *Ophiopogon jaburan* | MT555151 |
| 49 | Asparagaceae | *Oziroe biflora* | KX931463 |
| 50 | Asparagaceae | *Polygonatum acuminatifolium* | MZ150867 |
| 51 | Asparagaceae | *Polygonatum cirrhifolium* | MZ029092 |
| 52 | Asparagaceae | *Polygonatum curvistylum* | MZ150837 |
| 53 | Asparagaceae | *Polygonatum filipes* | OL405010 |
| 54 | Asparagaceae | *Polygonatum hirtum* | MZ150844 |
| 55 | Asparagaceae | *Polygonatum hookeri* | MZ150845 |
| 56 | Asparagaceae | *Polygonatum hunanense* | MW373519 |
| 57 | Asparagaceae | *Polygonatum inflatum* | MZ150850 |
| 58 | Asparagaceae | *Polygonatum involucratum* | MZ150852 |
| 59 | Asparagaceae | *Polygonatum jinzhaiense* | MZ150853 |
| 60 | Asparagaceae | *Polygonatum macropodum* | NC_058562 |
| 61 | Asparagaceae | *Polygonatum mengtzense* | OL587680 |
| 62 | Asparagaceae | *Polygonatum multiflorum* | MZ150855 |
| 63 | Asparagaceae | *Polygonatum nodosum* | MZ150857 |
| 64 | Asparagaceae | *Polygonatum odoratum* | MZ150859 |
| 65 | Asparagaceae | *Polygonatum prattii* | MZ150860 |
| 66 | Asparagaceae | *Polygonatum punctatum* | MZ150861 |
| 67 | Asparagaceae | *Polygonatum sibiricum* | MW373521 |
| 68 | Asparagaceae | *Polygonatum stewartianum* | OL405018 |
| 69 | Asparagaceae | *Polygonatum tessellatum* | MZ150863 |
| 70 | Asparagaceae | *Polygonatum uncinatum* | MZ150864 |
| 71 | Asparagaceae | *Polygonatum zanlanscianense* | MW800891 |
| 72 | Asparagaceae | *Reineckea carnea* | MK801116 |
| 73 | Asparagaceae | *Rohdea chinensis* | MH356725 |
| 74 | Asparagaceae | *Rohdea tonkinensis* | MW822041 |
| 75 | Asparagaceae | *Yucca queretaroensis* | KX931468 |
| 76 | Asphodelaceae | *Aloe maculata* | KX377523 |
| 77 | Asphodelaceae | *Eremurus robustus* | MN315570 |
| 78 | Asphodelaceae | *Xanthorrhoea preissii* | KX822774 |
| 79 | Colchicaceae | *Colchicum autumnale* | KP125337 |
| 80 | Colchicaceae | *Disporum sessile* | MN332241 |
| 81 | Hypoxidaceae | *Molineria capitulata* | MT610372 |
| 82 | Iridaceae | *Iris domestica* | MT001880 |
| 83 | Iridaceae | *Iris lactea* var. *lactea* | MT740331 |
| 84 | Iridaceae | *Iris sanguinea* | KT626943 |
| 85 | Liliaceae | *Lilium fargesii* | KX592156 |
| 86 | Liliaceae | *Lilium henryi* | KY748302 |
| 87 | Melanthiaceae | *Paris delavayi* | MN125581 |
| 88 | Melanthiaceae | *Paris fargesii* | MN125595 |
| 89 | Orchidaceae | *Bletilla striata* | MT193723 |
| 90 | Orchidaceae | *Neuwiedia singapureana* | KM244735 |
| 91 | Orchidaceae | *Oreorchis patens* | MN200369 |
| 92 | Orchidaceae | *Pholidota articulata* | MT712149 |
