# Supplementary material for: Phylogeny and evolution of Asparagaceae subfamily Nolinoideae: new insights from plastid phylogenomics
Source: Ann Bot. 2022 Nov 26;131(2):301–12. doi: 10.1093/aob/mcac144 (PMC9992941; doi:10.1093/aob/mcac144)
Supplement: mcac144_suppl_Supplementary_Table_S3 [file mcac144_suppl_supplementary_table_s3.docx]

**Table S3.** Summary of Illumina sequence and plastome assembly.

| Taxon | No. of clean reads | Size of plastid genome (bp) | No. of mapped reads | Coverage (×) |
| --- | --- | --- | --- | --- |
| *Polygonatum humile* (Ji Y 2018115) | 155,312 | 10,660,910 | 573,219 | 547.368 |
| *Polygonatum franchetii* (Ji Y 2018222) | 155,607 | 7,835,376 | 214,203 | 170.324 |
| *Polygonatum kingianum* (Ji Y 2019003) | 155,687 | 12,040,222 | 135,372 | 128.963 |
| *Polygonatum cyrtonema* (Ji Y 2019077) | 155,521 | 21,033,996 | 728,340 | 500.297 |
| *Rohdea yunnanensis* (Ji Y 2019086) | 156,934 | 14,427,494 | 320,264 | 285.652 |
| *Rohdea chinensis* (Ji Y 2019087) | 157,102 | 48,456,928 | 1,327,423 | 1256.114 |
| *Ophiopogon chingii* (Ji Y 2019088) | 156,918 | 20,322,130 | 476,140 | 433.127 |
| *Liriope muscari* (2) (Ji Y 2019091) | 157,060 | 15,905,064 | 119,202 | 113.727 |
| *Aspidistra cavicola* (Ji Y 2019092) | 156,385 | 16,331,810 | 1,131,630 | 1065.437 |
| *Aspidistra yingjiangensis* (Ji Y 2019093) | 156,413 | 20,024,784 | 524,009 | 418.056 |
| *Speirantha gardenii* (1) (Ji Y 2019094) | 156,874 | 13,364,630 | 816,686 | 784.013 |
| *Reineckea carnea* (1) (Ji Y 2019101) | 157,059 | 14,761,946 | 271,265 | 227.339 |
| *Liriope muscari* (1) (Ji Y 2019107) | 157,060 | 15,044,308 | 133,580 | 128.565 |
| *Disporopsis aspersa* (Ji Y 2019108) | 156,137 | 18,499,256 | 275,789 | 265.889 |
| *Rohdea longipedunculata* (Ji Y 2019109) | 156,932 | 20,697,504 | 591,831 | 514.875 |
| *Ophiopogon bodinieri* (An H 2019110) | 156,762 | 16,394,642 | 348,988 | 334.314 |
| *Ophiopogon japonicus* (An H 2019111) | 157,195 | 14,748,836 | 156,102 | 147.965 |
| *Reineckea carnea* (2) (An H 2019112) | 157,014 | 19,409,302 | 725,399 | 695.245 |
| *Maianthemum bifolium* (Ji Y 2018163) | 156,961 | 20,911,482 | 371,491 | 355.882 |
| *Convallaria majalis* (3) (Ji Y 2018168) | 162,102 | 19,918,684 | 507,237 | 485.741 |
| *Maianthemum japonicum* (An H 2019002) | 157,163 | 34,342,810 | 719,492 | 696.265 |
| *Polygonatum verticillatum* (Yi S 2019128) | 155,505 | 33,173,560 | 133,429 | 132.342 |
| *Ruscus aculeatus* (Liu C 2020049) | 153,883 | 28,813,500 | 523,406 | 506.526 |
| *Dracaena trifasciata* (Jin L 2020051) | 155,180 | 30,083,200 | 1,066,218 | 1031.8 |
| *Tupistra grandistigma* (Ji Y 2020111) | 157,036 | 28,768,946 | 141,493 | 139.864 |
| *Disporopsis fuscopicta* (Liu C et al 12CS4462) | 156,000 | 23,222,912 | 349,613 | 338.111 |
| *Aspidistra obliquipeltata* (B2013-477) | 156,415 | 20,420,304 | 111,418 | 107.562 |
| *Convallaria majalis* (1) (Liu M et al 598) | 162,226 | 18,429,646 | 218,711 | 203.732 |
| *Speirantha gardenii* (2) (Ya J 15CS11175) | 156,776 | 22,084,868 | 173,288 | 170.544 |
| *Asparagus officinalis* (Ji Y 2019084) | 156,786 | 9,426,324 | 493,403 | 367.165 |
| *Asparagus schoberioides* (Ji Y 2018152) | 156,876 | 16,394,642 | 350,176 | 334.679 |
| *Theropogon pallidus* (Exp. 4213) | 156,577 | 84,249,714 | 445,501 | 429.633 |
| *Peliosanthes macrostegia* (LED9297) | 156,707 | 8,557,760 | 124,036 | 131.715 |
| *Beaucarnea recurvata* (Luo Y s. n.) | 155,953 | 17,798,362 | 312,173 | 302.195 |
| *Rohdea japonica* (Dong A et al Tancm966) | 156,907 | 22,320,152 | 365,429 | 349.162 |
| *Rohdea delavayi* (15CS10509) | 156,855 | 18,284,280 | 202,762 | 194.968 |
| *Convallaria majalis* (2) (Zhou H 1506) | 162,227 | 20,017,206 | 535,009 | 497.093 |
| *Rohdea aurantiaca* (Zhou Y 141) | 156,927 | 14,160,520 | 152,939 | 147.06 |
| *Tupistra muricata* (13CS6063) | 157,063 | 14,274,708 | 130,253 | 129.09 |
