# Supplementary material for: Phylogeny and evolution of Asparagaceae subfamily Nolinoideae: new insights from plastid phylogenomics
Source: Ann Bot. 2022 Nov 26;131(2):301–12. doi: 10.1093/aob/mcac144 (PMC9992941; doi:10.1093/aob/mcac144)
Supplement: mcac144_suppl_Supplementary_Table_S4 [file mcac144_suppl_supplementary_table_s4.docx]

**Table S4. Features of Newly sequenced Nolinoideae Plastomes.**

| **Taxa** | **Voucher**  **(GenBank accession)** | **Plastome** | | **LSC** | | **SSC** | | **IR** | | **Coding sequences** | | **Uncoding sequences** | |
| --- | --- | --- | --- | --- | --- | --- | --- | --- | --- | --- | --- | --- | --- |
|  |  | **Size**  **(bp)** | **GC**  **(%)** | **Size**  **(bp)** | **GC**  **(%)** | **Size**  **(bp)** | **GC (%)** | **Size**  **(bp)** | **GC (%)** | **Size**  **(bp)** | **GC**  **(%)** | **Size**  **(bp)** | **GC**  **(%)** |
| *Aspidistra cavicola* | Ji Y 2019092 | 156,385 | 37.6 | 85,317 | 35.6 | 18,220 | 31.6 | 26,424 | 43 | 90,522 | 40.3 | 65,863 | 33.9 |
| *Aspidistra obliquipeltata* | B2013-477 | 156,415 | 37.6 | 85,377 | 35.6 | 18,200 | 31.7 | 26,419 | 43 | 90,495 | 40.3 | 65,920 | 34 |
| *Aspidistra yingjiangensis* | Ji Y 2019093 | 156,413 | 37.6 | 85,174 | 35.6 | 18,213 | 31.6 | 26,513 | 43 | 90,325 | 40.3 | 66,088 | 34 |
| *Beaucarnea recurvata* | Ji Y 2021001 | 155,953 | 37.6 | 84,540 | 35.6 | 18,597 | 31.4 | 26,408 | 43 | 90,623 | 40.3 | 65,330 | 34 |
| *Convallaria keiskei* | MH680946 | 162,109 | 37.9 | 85,344 | 35.7 | 18,487 | 31.4 | 29,139 | 43.2 | 90,417 | 40.3 | 71,692 | 34.9 |
| *Convallaria majalis* (1) | Liu M et al 598 | 162,226 | 37.9 | 85,425 | 35.6 | 18,495 | 31.4 | 29,153 | 43.2 | 90,326 | 40.3 | 71,900 | 34.8 |
| *Convallaria majalis* (2) | Ji Y 2018168 | 162,102 | 37.9 | 85,301 | 35.6 | 18,495 | 31.4 | 29,153 | 43.2 | 90,326 | 40.3 | 71,776 | 34.8 |
| *Convallaria majalis* (3) | Zhou H 1506 | 162,227 | 37.6 | 85,426 | 35.6 | 18,495 | 31.4 | 29,153 | 43.2 | 90,326 | 40.3 | 71,901 | 34.8 |
| *Disporopsis aspersa* | Ji Y 2019108 | 156,137 | 37.7 | 85,044 | 35.7 | 18,539 | 31.6 | 26,277 | 43.1 | 90,441 | 40.3 | 65,696 | 34.1 |
| *Disporopsis fuscopicta* | Liu C et al 12CS4462 | 156,000 | 37.7 | 84,965 | 35.7 | 18,551 | 31.6 | 26,242 | 43.1 | 90,678 | 40.3 | 65,322 | 34.1 |
| *Dracaena draco* | MN990038 | 155,422 | 37.6 | 83,944 | 35.6 | 18,472 | 31.2 | 26,504 | 42.9 | 90,252 | 40.2 | 65,170 | 34 |
| *Dracaena hokouensis* | MN200197 | 155,340 | 37.5 | 83,796 | 35.5 | 18,494 | 31.2 | 26,525 | 42.9 | 90,376 | 40.2 | 64,964 | 33.8 |
| *Dracaena terniflora* | MN200198 | 155,347 | 37.5 | 83,794 | 35.5 | 18,493 | 31.2 | 26,530 | 42.9 | 90,375 | 40.2 | 64,972 | 33.9 |
| *Dracaena trifasciata* | Jin L 2020051 | 155,180 | 37.5 | 83,681 | 35.4 | 18,473 | 31.1 | 26,513 | 42.9 | 90,273 | 40.1 | 64,907 | 33.7 |
| *Heteropolygonatum altelobatum* | MH891734 | 155,548 | 37.6 | 84,701 | 35.6 | 18,361 | 31.5 | 26,243 | 42.9 | 90,331 | 40.2 | 65,217 | 34 |
| *Heteropolygonatum marmoratum* | MH891735 | 155,516 | 37.6 | 84,627 | 35.6 | 18,369 | 31.6 | 26,260 | 43 | 90,458 | 40.2 | 65,058 | 34 |
| *Heteropolygonatum pendulum* | MH891736 | 155,438 | 37.6 | 84,609 | 35.7 | 18,365 | 31.7 | 26,232 | 43 | 90,335 | 40.3 | 65,103 | 34.1 |
| *Liriope muscari* | MK210628 | 156,754 | 37.6 | 85,118 | 35.6 | 18,680 | 31.3 | 26,478 | 43 | 89,993 | 40.3 | 66,761 | 34 |
| *Liriope muscari* (1) | Ji Y 2019107 | 157,060 | 37.6 | 85,380 | 35.6 | 18,712 | 31.3 | 26,484 | 43 | 90,527 | 40.3 | 66,533 | 34 |
| *Liriope muscari* (2) | Ji Y 2019091 | 157,060 | 37.6 | 85,380 | 35.6 | 18,712 | 31.3 | 26,484 | 43 | 90,527 | 40.3 | 66,533 | 34 |
| *Liriope spicata* | MH680945 | 157,055 | 37.6 | 85,374 | 35.6 | 18,727 | 31.2 | 26,477 | 43 | 90,694 | 40.3 | 66,428 | 34 |
| *Maianthemum bicolor* | KX790362 | 157,176 | 37.6 | 85,621 | 35.6 | 18,394 | 31.5 | 26,542 | 43 | 90,743 | 40.3 | 66,500 | 34 |
| *Maianthemum bifolium* | Ji Y 2018163 | 156,961 | 37.6 | 85,604 | 35.5 | 18,421 | 31.6 | 26,468 | 43 | 90,717 | 40.3 | 66,244 | 33.9 |
| *Maianthemum dilatatum* | MF150041 | 156,921 | 37.6 | 85,554 | 35.6 | 18,431 | 31.6 | 26,468 | 43 | 85,991 | 40.9 | 70,930 | 33.5 |
| *Maianthemum japonicum* | An H 2019002 | 157,163 | 37.5 | 85,706 | 35.5 | 18,451 | 31.4 | 26,503 | 42.9 | 90,637 | 40.2 | 66,526 | 33.8 |
| *Nolina atopocarpa* | KX931462 | 156,792 | 37.6 | 85,274 | 35.6 | 18,578 | 31.4 | 26,470 | 43 | 90,462 | 40.3 | 66,330 | 34 |
| *Ophiopogon bodinieri* | An H 2019110 | 156,762 | 37.7 | 84,988 | 35.8 | 18,664 | 31.4 | 26,555 | 42.9 | 90,743 | 40.3 | 66,019 | 34.1 |
| *Ophiopogon chingii* | Ji Y 2019088 | 156,918 | 37.7 | 85,280 | 35.7 | 18,676 | 31.5 | 26,481 | 43 | 90,719 | 40.3 | 66,199 | 34 |
| *Ophiopogon japonicus* | An H 2019111 | 157,195 | 37.6 | 85,513 | 35.7 | 18,738 | 31.4 | 26,472 | 43 | 90,758 | 40.3 | 66,437 | 33.9 |
| *Ophiopogon japonicus* | MK952744 | 156,679 | 37.7 | 84,925 | 35.7 | 18,036 | 31.7 | 26,852 | 42.8 | 90,680 | 40.3 | 65,999 | 34.1 |
| *Peliosanthes macrostegia* | LED9297 | 156,707 | 37.6 | 85,587 | 35.7 | 18,504 | 31.4 | 26,308 | 43.1 | 90,607 | 40.3 | 66,100 | 34 |
| *Polygonatum cyrtonema* | Ji Y 2019077 | 155,521 | 37.7 | 84,461 | 35.7 | 18,292 | 31.7 | 26,384 | 42.9 | 90,373 | 40.3 | 65,148 | 34.1 |
| *Polygonatum franchetii* | Ji Y 2018222 | 155,607 | 37.7 | 84,437 | 35.7 | 18,426 | 31.5 | 26,372 | 42.9 | 90,370 | 40.2 | 65,237 | 34.1 |
| *Polygonatum humile* | Ji Y 2018115 | 155,312 | 37.7 | 84,410 | 35.7 | 18,468 | 31.5 | 26,217 | 43.0 | 90,207 | 40.3 | 65,105 | 34.1 |
| *Polygonatum kingianum* | Ji Y 2019003 | 155,687 | 37.7 | 84,771 | 35.7 | 18,530 | 31.6 | 26,193 | 43.0 | 90,290 | 40.3 | 65,397 | 34.1 |
| *Polygonatum verticillatum* | Yi S 2019128 | 155,505 | 37.7 | 84,207 | 35.7 | 18,468 | 31.5 | 26,415 | 42.9 | 90,600 | 40.2 | 64,958 | 34.1 |
| *Reineckea carnea* | MK801116 | 157,059 | 37.6 | 85,474 | 35.6 | 18,535 | 31.5 | 26,525 | 43 | 90,665 | 40.3 | 66,394 | 34 |
| *Reineckea carnea* (1) | Ji Y 2019101 | 157,059 | 37.6 | 85,472 | 35.6 | 18,535 | 31.5 | 26,526 | 43 | 90,604 | 40.3 | 66,455 | 34 |
| *Reineckea carnea* (2) | An H 2019112 | 157,014 | 37.6 | 85,426 | 35.6 | 18,536 | 31.4 | 26,526 | 43 | 90,666 | 40.3 | 66,348 | 34 |
| *Rohdea aurantiaca* | Zhou Y 141 | 156,927 | 37.6 | 85,628 | 35.6 | 18,557 | 31.4 | 26,371 | 43 | 90,360 | 40.3 | 66,567 | 33.9 |
| *Rohdea* *chinensis* | Ji Y 2019087 | 157,102 | 37.6 | 85,446 | 35.6 | 18,620 | 31.4 | 26,518 | 43 | 90,577 | 40.3 | 66,525 | 33.9 |
| *Rohdea chinensis* | MH356725 | 158,795 | 37.2 | 86,524 | 35.1 | 21,363 | 31.2 | 25,340 | 43.3 | 91,190 | 40.2 | 67,605 | 33.1 |
| *Rohdea delavayi* | 15CS10509 | 156,855 | 37.6 | 85,339 | 35.6 | 18,600 | 31.3 | 26,458 | 43 | 90,394 | 40.3 | 66,461 | 33.9 |
| *Rohdea japonica* | Dong A et al Tancm966 | 156,907 | 37.6 | 85,391 | 35.6 | 18,600 | 31.3 | 26,458 | 43 | 90,394 | 40.3 | 66,513 | 33.9 |
| *Rohdea longipedunculata* | Ji Y 2019109 | 156,932 | 37.6 | 85,354 | 35.6 | 18,554 | 31.4 | 26,512 | 43 | 90,634 | 40.3 | 66,298 | 34 |
| *Rohdea yunnanensis* | Ji Y 2019086 | 156,934 | 37.6 | 85,566 | 35.6 | 18,554 | 31.4 | 26,407 | 43 | 90,345 | 40.3 | 66,589 | 34 |
| *Ruscus aculeatus* | Liu C 2020049 | 153,883 | 37.8 | 83,007 | 35.9 | 18,354 | 31.5 | 26,261 | 43.1 | 89,869 | 40.3 | 64,014 | 34.4 |
| *Speirantha gardenii* (1) | Ji Y 2019094 | 156,874 | 37.6 | 85,363 | 35.6 | 18,637 | 31.5 | 26,437 | 43 | 90,660 | 40.3 | 66,240 | 34 |
| *Speirantha gardenii* (2) | Ya J 15CS11175 | 156,776 | 37.6 | 85,267 | 35.6 | 18,635 | 31.5 | 26,437 | 43 | 90,601 | 40.3 | 66,175 | 34 |
| *Theropogon pallidus* | Exp. 4213 | 156,577 | 37.7 | 85,605 | 35.6 | 18,205 | 31.5 | 26,436 | 43 | 90,168 | 40.3 | 66,409 | 34.1 |
| *Tupistra grandistigma* | Ji Y 2020111 | 157,036 | 37.5 | 85,482 | 35.4 | 18,526 | 31.4 | 26,514 | 43 | 90,295 | 40.3 | 66,741 | 33.8 |
| *Tupistra muricata* | 13CS6063 | 157,063 | 37.6 | 85,489 | 35.4 | 18,546 | 31.4 | 26,514 | 43 | 90,295 | 40.3 | 66,768 | 33.8 |
