# Supplementary material for: Phylogeny and evolution of Asparagaceae subfamily Nolinoideae: new insights from plastid phylogenomics
Source: Ann Bot. 2022 Nov 26;131(2):301–12. doi: 10.1093/aob/mcac144 (PMC9992941; doi:10.1093/aob/mcac144)
Supplement: mcac144_suppl_Supplementary_Table_S5 [file mcac144_suppl_supplementary_table_s5.docx]

| **Category of Genes** | **Group of gene** | **Name of gene** |
| --- | --- | --- |
| Self-replication | Ribosomal RNA genes | *rrn4.5*×2, *rrn5*×2, *rrn16*×2, *rrn23*×2 |
|  | Transfer RNA genes | *trnC-GCA*, *trnD-GUC*, *trnE-UUC*, *trnF-GAA*, *trnG-GCC*, *trnG-UCC**, *trnH-GUG*, *trnK-UUU**, *trnL-UAA**, *trnL-UAG*, *trnM-CAU*, *trnP-UGG*, *trnQ-UUG*, *trnR-UCU*, *trnR-ACG*, *trnS-GCU*, *trnS-GGA*, *trnS-UGA*, *trnT-UGU*, *trnT-GGU*, *trnV-UAC**, *trnY-GUA*, *trnW-CCA*, *trnfM-CAU*, *trnA-UGC**×2, *trnI-CAU*×2, *trnI-GAU**×2, *trnL-CAA*×2, *trnN-GUU*×2, *trnR-ACG*×2, *trnV-GAC*×2 |
|  | Ribosomal protein (small subunit) | *rps2*, *rps3*, *rps4*, *rps7*×2, *rps8*, *rps11*, *rps12***×2, *rps14*, *rps15*, *rps16**, *rps18*, *rps19* |
|  | Ribosomal protein (large subunit) | *rpl2*×2, *rpl14*, *rpl16**, *rpl20*, *rpl22*, *rpl23*×2, *rpl32*, *rpl33*, *rpl36* |
|  | RNA polymerase | *rpoA*, *rpoB*, *rpoC1**, *rpoC2* |
|  | Translational initiation factor | *infA^#^* |
| Genes for photosynthesis | Subunits of photosystem I | *psaA*, *psaB*, *psaC*, *psaI*, *psaJ*, *ycf3***, *ycf4* |
|  | Subunits of photosystem II | *psbA*, *psbB*, *psbC*, *psbD*, *psbE*, *psbF*, *psbH*, *psbI*, *psbJ*, *psbK*, *psbL*, *psbM*, *psbN*, *psbT*, *psbZ* |
|  | Subunits of cytochrome | *petA*, *petB**, *petD**, *petG*, *petL*, *petN* |
|  | Subunits of ATP synthase | *atpA*, *atpB*, *atpE*, *atpF**, *atpH*, *atpI* |
|  | Large subunit of Rubisco | *rbcL* |
|  | Subunits of NADH dehydrogenase | *ndhA**, *ndhB**×2, *ndhC*, *ndhD*, *ndhE*, *ndhF*, *ndhG*, *ndhH*, *ndhI*, *ndhJ*, *ndhK* |
| Other genes | Maturase | *matK* |
|  | Envelope membrane protein | *cemA* |
|  | Subunit of acetyl-CoA | *accD* |
|  | Synthesis gene | *ccsA* |
|  | ATP-dependent protease | *clpP*** |
|  | Component of TIC complex | *ycf1*×2 |
| Genes of unknown function | Conserved open reading frames | *ycf2*×2, *ycf15^#^*×2 |

**Table S5.** List of genes identified in Nolinoideae plastomes.

×2: Two gene copies in IR regions; *: With one intron; **: With two introns; ^#^: Pseudogene.
