# Supplementary material for: Phylogeny and evolution of Asparagaceae subfamily Nolinoideae: new insights from plastid phylogenomics
Source: Ann Bot. 2022 Nov 26;131(2):301–12. doi: 10.1093/aob/mcac144 (PMC9992941; doi:10.1093/aob/mcac144)
Supplement: mcac144_suppl_Supplementary_Table_S6 [file mcac144_suppl_supplementary_table_s6.docx]

**Table S6. Sequence characteristics of 68 protein-coding genes (PCGs) involved in the phylogenetic analyses.**

| **Gene** | **Aligned length (bp)** | **No. of variable sites (divergence, %)** | **No. of parsimony informative sites (divergence, %)** |
| --- | --- | --- | --- |
| *atpA* | 1,518 | 453 (29.84) | 332 (21.87) |
| *atpB* | 1,500 | 435 (29.00) | 319 (21.27) |
| *atpE* | 402 | 152 (37.81) | 103 (25.62) |
| *atpF* | 576 | 243 (42.19) | 160 (27.78) |
| *atpH* | 246 | 52 (21.14) | 36 (14.63) |
| *atpI* | 744 | 193 (25.94) | 138 (18.55) |
| *matK* | 1,694 | 929 (54.84) | 697 (41.15) |
| *ndhA* | 1,104 | 442 (40.04) | 327 (29.62) |
| *ndhB* | 1,512 | 190 (12.57) | 86 (5.69) |
| *ndhC* | 363 | 95 (26.17) | 70 (19.28) |
| *ndhD* | 1,381 | 543 (39.32) | 481 (34.83) |
| *ndhE* | 306 | 94 (30.72) | 78 (25.49) |
| *ndhF* | 2,276 | 1017 (44.68) | 767 (233.70) |
| *ndhH* | 1,185 | 398 (33.59) | 311 (26.24) |
| *ndhI* | 516 | 181 (35.08) | 136 (26.36) |
| *ndhJ* | 477 | 147 (30.82) | 103 (21.59) |
| *ndhK* | 664 | 205 (30.87) | 151 (22.74) |
| *petA* | 972 | 296 (30.45) | 209 (21.50) |
| *petB* | 648 | 155 (23.92) | 112 (17.28) |
| *petD* | 474 | 108 (22.78) | 82 (17.30) |
| *petG* | 114 | 28 (24.56) | 19 (16.67) |
| *petL* | 96 | 28 (29.17) | 18 (18.75) |
| *petN* | 90 | 19 (21.11) | 13 (14.44) |
| *psaA* | 2,254 | 519 (23.03) | 377 (16.73) |
| *psaB* | 2,205 | 479 (21.72) | 349 (15.83) |
| *psaC* | 246 | 57 (23.17) | 45 (18.29) |
| *psaI* | 111 | 36 (32.43) | 26 (23.42) |
| *psaJ* | 126 | 33 (26.19) | 24 (19.05) |
| *psbA* | 1,062 | 227 (21.37) | 165 (15.54) |
| *psbB* | 1,527 | 376 (24.62) | 288 (18.86) |
| *psbC* | 1,503 | 335 (22.29) | 249 (16.57) |
| *psbD* | 1,062 | 221 (20.81) | 171 (16.10) |
| *psbE* | 252 | 49 (19.44) | 31 (12.30) |
| *psbF* | 120 | 21 (17.50) | 15 (12.50) |
| *psbH* | 231 | 70 (30.30) | 58 (25.11) |
| *psbI* | 111 | 29 (26.13) | 18 (16.22) |
| *psbJ* | 123 | 29 (23.58) | 22 (17.89) |
| *psbK* | 204 | 84 (41.18) | 56 (27.45) |
| *psbL* | 117 | 15 (12.82) | 10 (8.55) |
| *psbM* | 105 | 30 (28.57) | 22 (20.95) |
| *psbN* | 132 | 34 (25.76) | 24 (18.18) |
| *psbT* | 102 | 20 (19.61) | 15 (14.71) |
| *psbZ* | 189 | 39 (20.63) | 28 (14.81) |
| *rbcL* | 1437 | 432 (30.06) | 332 (23.10) |
| *rpl2* | 828 | 149 (18.00) | 87 (10.51) |
| *rpl14* | 369 | 119 (32.25) | 84 (22.76) |
| *rpl16* | 366 | 146 (39.89) | 113 (30.87) |
| *rpl20* | 368 | 176 (47.83) | 120 (32.61) |
| *rpl22* | 386 | 189 (48.96) | 136 (35.23) |
| *rpl33* | 210 | 91 (43.33) | 63 (30.00) |
| *rpl36* | 114 | 43 (37.72) | 27 (23.68) |
| *rpoA* | 936 | 397 (42.41) | 266 (28.42) |
| *rpoB* | 3,246 | 1102 (33.95) | 735 (22.64) |
| *rpoC1* | 1,380 | 466 (33.77) | 322 (23.33) |
| *rpoC2* | 4,229 | 1848 (43.70) | 1322 (31.26) |
| *rps3* | 699 | 298 (42.63) | 219 (31.33) |
| *rps4* | 609 | 219 (35.96) | 140 (22.99) |
| *rps7* | 468 | 86 (18.38) | 39 (8.33) |
| *rps8* | 417 | 193 (46.28) | 119 (28.54) |
| *rps11* | 435 | 190 (43.68) | 128 (29.43) |
| *rps12* | 383 | 95 (24.80) | 37 (9.66) |
| *rps14* | 305 | 106 (34.75) | 68 (22.30) |
| *rps15* | 279 | 142 (50.90) | 107 (38.35) |
| *rps18* | 372 | 102 (27.42) | 47 (12.63) |
| *ycf1* | 7,953 | 3762 (47.30) | 2607 (32.78) |
| *ycf2* | 8,358 | 2210 (26.44) | 1024 (12.25) |
| *ycf3* | 516 | 120 (23.26) | 91 (17.64) |
| *ycf4* | 555 | 216 (38.92) | 157 (28.29) |
| Total | 65,859 | 21994 (33.40) | 15068 (22.88) |
